# Exsolution of Pt Nanoparticles from Mixed Zr/Gd‐CeO_2_ Oxides for Microbial Fuel Cell‐Based Biosensors

**DOI:** 10.1002/smsc.202400619

**Published:** 2025-04-01

**Authors:** Alex Martinez Martin, Shailza Saini, Anna Salvian, Tarique Miah, Cheuk Yiu Chan, Claudio Avignone Rossa, Siddharth Gadkari, Kalliopi Kousi

**Affiliations:** ^1^ School of Chemistry and Chemical Engineering University of Surrey Guildford GU2 7XH UK; ^2^ Department of Microbial Sciences University of Surrey Guildford GU2 7XH UK

**Keywords:** biosensors, CeO_2_, exsolution, microbial fuel cells, platinum catalysts

## Abstract

Cerium oxide (CeO_2_) is a widely used catalyst support in electrochemical and catalytic applications due to its ability to form oxygen vacancies and strong metal‐support interactions. However, conventionally prepared CeO_2_ catalysts often suffer from deactivation due to sintering and poisoning. Incorporating dopants such as gadolinium (Gd) and zirconium (Zr) into its lattice improves oxygen ion mobility, thermal stability, and resistance to poisoning. Platinum (Pt) is a commonly used catalyst for the oxygen reduction reaction in microbial fuel cells for real‐time biochemical oxygen demand monitoring. However, its high cost, scarcity, and susceptibility to fouling and poisoning limit implementation in wastewater treatment plants. This study employs the exsolution method to investigate the formation of Pt nanoparticles from undoped, Zr‐, and Gd‐doped CeO_2_ matrices. It is shown that the Gd‐doped matrix exhibits the optimal particle characteristics, while electrochemical evaluation in the microbial fuel cells also reveals that it outperforms the other studied materials, in terms of sensitivity and stability. By integrating exsolution with dopant engineering, in an innovative approach, we lower costs, maintain performance, and enhance the operational stability of the cathode material, paving the way for cost‐effective and sustainable applications in biosensing but also other catalytic applications of interest.

## Graphical abstract

By combining exsolution with dopant engineering, in an innovative approach, mixed cerium oxides with gadolinium and zirconium enhance the stability and performance of a platinum‐based microbial fuel cell. This approach offers cost‐effective, sustainable solutions for biosensing and array of other catalytic applications.

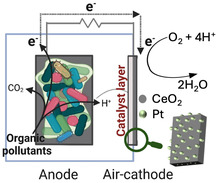

## 1 Introduction

Simple oxides have been widely utilized as supports in catalysis due to their intrinsic stability and their ability to enhance the activity of catalytic metals. Their utility is further augmented by additional properties, such as the capacity to establish strong metal‐oxide interactions or to generate oxygen vacancies. These features are crucial in a variety of catalytic and electrochemical processes.^[^ ^1^ ^]^ In particular, cerium oxide (CeO_2_) is one of the most commonly used oxide supports in various catalytic reactions^[^ ^2^ ^]^ and electrochemical devices,^[^ ^3^, ^4^, ^5^ ^]^ as it is a versatile material that incorporates all the above characteristics. However, CeO_2_‐based catalysts still tend to suffer from thermal,^[^ ^6^ ^]^ oxidative,^[^ ^7^ ^]^ and chemical^[^ ^8^ ^]^ deactivation under reaction conditions. To overcome these problems, several dopants have been incorporated into the CeO_2_ lattice, such as Gd,^[^ ^9^ ^]^ Sm,^[^ ^9^, ^10^ ^]^ Zr,^[^ ^11^ ^]^ and La.^[^ ^12^ ^]^ The addition of Zr to the CeO_2_ core structure has been shown to increase the stability of the doped material in electrochemical processes,^[^ ^13^ ^]^ demonstrate superior oxygen capacity as compared to undoped CeO_2_, and enhance thermal resistance and catalytic reaction rates.^[^ ^14^ ^]^ The use of Gd as a dopant is particularly interesting, as it seems to promote the oxygen ions diffusion throughout the lattice while maintaining a stable structure,^[^ ^12^ ^]^ and is also known to increase its resistance to deactivation due to sulfur poisoning.^[^ ^15^ ^]^ Gd‐doped CeO_2_ also is widely known to increase the number of oxygen vacancies and ionic conductivity, making it suitable for use in a range of applications, such as electrocatalytic, biomedical, and optical processes.^[^ ^16^ ^]^

All of the above demonstrate that CeO_2_ and its mixed oxides are perfect candidates for use as supports in a large area of applications. However, support cannot magically resolve all challenges associated with catalyst design. For example, impregnated CeO_2_ and its mixed oxides have long been used as catalysts for soot oxidation,^[^ ^17^ ^]^ three‐way catalysts,^[^ ^18^ ^]^ CO_2_ methanation,^[^ ^19^ ^]^ and hydrogen and oxygen evolution reactions.^[^ ^20^ ^]^ These materials still often face limitations, hindering their operational lifespan. Specifically, typical cathodic materials, such as Pt‐based catalysts, tend to be expensive and suffer from a loss in activity due to deactivation.^[^ ^21^ ^]^ To overcome this deactivation issue, materials containing two or more active elements tend to be used. These materials are nonetheless limited due to their complex synthetic routes and increased prices.^[^ ^22^ ^]^ Common materials used as cathodes are activated carbon,^[^ ^23^ ^]^ graphene,^[^ ^24^ ^]^ and metal oxide catalysts.^[^ ^25^ ^]^ To enhance cathodic reaction rates, materials tend to be tailored by promoting the formation of vacancies, which favor these electrocatalytic reactions.^[^ ^26^, ^27^ ^]^ However, the development of active materials with oxygen vacancies that do not undergo quick deactivation still remains a challenge today. For example, efforts have been dedicated in recent years to developing suitable and cost‐effective cathodes for microbial fuel cells (MFCs), as cathodes currently account for 50% of the total cost of MFC systems.^[^ ^28^ ^]^

An MFC is a device that harnesses bacteria to convert organic and inorganic matter into electrical energy. MFCs have diverse applications, including removal of organic pollutants from soil and water,^[^ ^29^ ^]^ desalinization at large scale,^[^ ^30^ ^]^ and biosensing of pollutants in wastewater. The use of MFCs as biosensors for detecting total organic compounds in wastewater, commonly referred to as biological oxygen demand (BOD), is particularly valuable because there is currently no simple, fast, and reliable method for BOD detection that operates without manual intervention. In an MFC, microbes form a biofilm on the surface of the anode, where they oxidize organic and inorganic compounds, transferring electrons from the bacterial cells to the anode material and generating electrical current in a closed circuit.^[^ ^31^, ^32^ ^]^ These electrons travel from the anode to the cathode through a current collector connected to an external resistor. At the cathode, a reduction reaction occurs in which electrons are used to reduce a molecule, typically oxygen, which combines with protons generated by bacterial activity to form water in a process called “oxygen reduction reaction” (ORR).

In recent years, the exsolution method has gained increased popularity given its potential to produce stable catalysts whilst maintaining high reaction rates.^[^ ^33^, ^34^ ^]^ In exsolution, upon H_2_ treatment of the solid, the initial oxide matrix is partially reduced, and reducible cations are exsolved onto the oxide's surface as nanoparticles, enhancing their dispersion and stability reactivity.^[^ ^33^ ^]^ Exsolution has proven to be more effective than other traditional catalytic methods such as impregnation, leading to higher reaction rates,^[^ ^35^, ^36^, ^37^ ^]^ weaker or less stable nanoparticles, or poor stability,^[^ ^38^, ^39^ ^]^ due to the embedded nature of the formed nanoparticles. Moreover, exsolution of nanoparticles has also demonstrated resistance to degradation and deactivation mechanisms.^[^ ^40^, ^41^ ^]^ Hence, thanks to their unique properties, exsolution of nanoparticles has been widely used in several fields, such as catalytic processes,^[^ ^39^ ^]^ chemical looping,^[^ ^42^ ^]^ solid oxide fuel cells,^[^ ^43^ ^]^ or co‐electrolytic processes.^[^ ^44^ ^]^ The exsolution of nanoparticles from fluorite‐type structure has not been studied as extensively as perovskites due to the limited cation ion mobility and tailorability.^[^ ^34^ ^]^ The potential of exsolution from non‐perovskite materials is rarely explored in the literature.^[^ ^45^ ^]^ However, these studies already demonstrate the feasibility of such structures for this concept.

Here, we probe the potential of fluorite‐based catalysts in MFCs via exsolution. We explore the effects of 2 wt% Pt‐substituted within a CeO_2_ matrix leveraging the principles of exsolution to promote the formation of anchored Pt nanoparticles. We also systematically compare the addition of dopants (Zr, Gd) and their impact on the morphology of a CeO_2_ matrix and the exsolution characteristics. We study how the electrocatalytic properties such as the formation of oxygen vacancies or the morphology of the materials are improved upon exsolution. Characterization techniques including Powder X‐ray diffraction (PXRD), scanning electron microscopy (SEM), and X‐ray photoelectron spectroscopy (XPS) were used to deconvolute the role of these dopants before and after exsolution. Given its high activity, corrosion resistance, and long‐term stability in electrocatalytic processes,^[^ ^46^ ^]^ the use of Pt as the active site is chosen here. This research aims to combine and harness Pt's high activity alongside undoped and Zr‐ and Gd‐doped CeO_2_'s ability to form oxygen vacancies and its enhanced resistance to the reaction conditions, ultimately improving the material´s electrocatalytic reaction rates. The combination of all these features is expected to promote ORR rates, significantly influencing the performance of our materials in MFCs.^[^ ^5^, ^47^ ^]^ Additionally, their effect on and in conjunction with exsolution has never been studied before. Ultimately, it is expected that the results of this research will also greatly impact other catalytic applications due to the potential of such catalysts in a plethora of processes.^[^ ^20^, ^48^ ^]^

## 2 Results and Discussion

In order to identify the optimal conditions that would best enable the incorporation and stability of Pt atoms, an initial assessment was conducted on the synthesis of CeO_2_. This initial screening concentrated on evaluating the crystal size and morphology of the synthesized oxide. Once the optimal conditions were identified for the Pt‐doped Pt_0.02_Ce_0.98_O_2_ (PCO), we then created materials that included Pt_0.02_Ce_0.8_Zr_0.18_O_2_ (PCZO) or Pt_0.0__2_Ce_0.8_Gd_0.18_O_2_ (PCGO) as dopants within the CeO_2_ matrix. Changes in the CeO_2_ matrix induced by the addition of these elements, such as the formation of oxygen vacancies or the formation of more stable structures, were further analyzed.

### 2.1 Initial Insights: Probing the Structure of Undoped and Pt‐Doped CeO_2_

**Figure 1:**
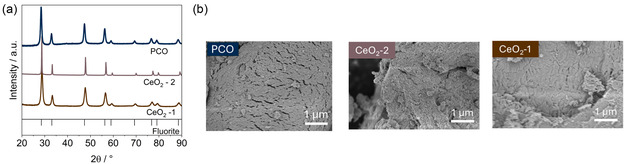
a) Undoped and Pt‐doped CeO_2_ characterization. PXRD pattern of CeO_2_ calcined at 600 °C for 5 h (CeO_2_‐1), calcined at 700 °C for 16 h (CeO_2_‐2), and Pt doped CeO_2_ calcined at 700 °C for 16 h (PCO). b) Microstructure images of the different materials. Left image corresponds to Pt‐doped CeO_2_ (PCO) calcined at 700 °C for 16 h. Middle image corresponds to CeO_2_ calcined at 700 °C for 16 h, denoted as CeO_2_‐2. Right image corresponds to CeO_2_ calcined at 600 °C for 6 h, denoted as CeO_2_‐1.

The purity and crystallinity of the prepared materials were analyzed using PXRD (**Figure** **1**a). The peaks displayed in the pattern correspond to a fluorite structure, indicating the intended synthesis of undoped CeO_2_.^[^ ^49^, ^50^ ^]^ As calcination temperature increased, fluorite peaks became narrower and more intense, indicating an increase in the crystal size of the oxide. In particular, the CeO_2_ average crystal size increased from 4.0 ± 0.4 nm when calcined at 600 °C for 6 h (referred as to CeO_2_‐1) up to 15.1 ± 1.0 nm when calcined at 700 °C for 16 h (referred as CeO_2_‐2). The microstructure of the cerium oxide matrix, evidenced by SEM analysis, is therefore also greatly affected by the calcination temperature, as expected. CeO_2_‐1 has a bulkier structure with no big pores observable. On the other hand, CeO_2_‐2 has a flake‐like structure and larger porosity, suggesting a potentially more reactive structure.^[^ ^51^ ^]^ Despite the fact that both samples could potentially be good candidates for our doping strategy, the higher surface area would promote mechanical stability and previously published research indicate that CeO_2_ synthesized at temperatures higher than 685 °C promoted the formation of oxygen vacancies within the material without altering the core structure.^[^ ^52^ ^]^ Therefore, the higher temperature was chosen, and further study of materials was conducted for species prepared at the latter conditions (700 °C for 16 h).

Incorporation of Pt did not significantly alter the material's initial crystallographic structure when calcining it at 700 °C for 16 h, as the fluorite CeO_2_ structure was preserved. Small amount of Pt was observed after calcination, evidenced by the Pt peak at 39.5°. This is not unlikely, as it has been previously reported that for noble metals such as Pt, exsolved particles have been detected, even under oxidizing conditions, after thermal treatment at 550 °C for 2 h.^[^ ^53^ ^]^ Therefore, we also calcined the same material (Pt‐doped CeO_2_) under 900 °C for 10 h, to probe for any reasonable amount of exsolution under such conditions. This actually resulted in complete decomposition of the Pt phase (see Figure S1, Supporting Information); hence, this sample was not further investigated.

Peak broadening was observed upon Pt‐doping in the initial CeO_2_ structure may suggest a decrease in the crystal size but could also be attributed to the lattice distortion induced by Pt incorporation.^[^ ^54^, ^55^ ^]^ The average crystal size of Pt doped CeO_2_ was also calculated using the Scherrer equation, obtaining a value of 4.9 ± 0.8 nm. This value was significantly lower than the undoped CeO_2_, which is attributed to the inhibition of crystal growth caused by Pt‐doping,^[^ ^56^ ^]^ which could be of benefit in this case since it has been observed that smaller crystal size tends to promote the exsolution of metallic nanoparticles.^[^ ^57^ ^]^

### 2.2 Zr‐ and Gd‐Doping of Pt‐CeO_2_ Catalysts

As detailed above, Zr and Gd are two of the most used dopants when it comes to increasing the performance of CeO_2_ for different reasons. Specifically, when Zr is doped in CeO_2_, no extrinsic oxygen vacancies are formed, as Zr^4+^ and Ce^4+^ are isovalent cations. However, due to the smaller size of Zr, it is expected that the matrix would counterbalance the increase of the cell unit upon reduction of Ce^4+^ to Ce^3+^, allowing for a higher degree of reduction of the CeO_2_ matrix, increasing the initial vacancies in this way.^[^ ^58^ ^]^ On the other hand, Gd‐doped CeO_2_ creates oxygen vacancies due to the charge imbalance generated (Gd^3+^ as compared to Ce^4+^)^[^ ^58^ ^]^ and is also known to increase ionic conductivity as compared to the undoped CeO_2_.^[^ ^59^ ^]^ The combination of all these features is expected to also promote ORR rates which is expected to heavily influence the performance of our materials when used in the MFCs.^[^ ^5^, ^47^ ^]^ Additionally, both systems have been widely used as fuel cell cathodes due to their excellent stability^[^ ^60^ ^]^ and high ionic conductivity when combined with CeO_2_ systems,^[^ ^61^ ^]^ but their effect on and in conjunction with exsolution has never been reported before.

**Figure 2:**
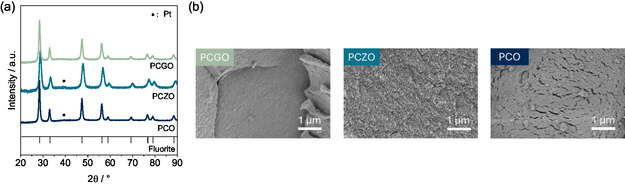
a) XRD patterns of the non‐modified, Zr‐ and Gd‐doped CeO_2_ after incorporating Pt within the structure. b) SEM images of the microstructure of the different Pt‐doped CeO_2_ matrices. Left image: PCGO. Middle image: PCZO. Right image: PCO.

The PXRD patterns of the synthesized catalysts are illustrated in **Figure** **2**, and phase identification suggests the formation of high purity materials. The absence of ZrO_2_ and Gd_2_O_3_ reflections in the patterns indicates the successful substitution of Ce cations by Gd or Zr. While no extra peaks were observed for PCGO, one peak at 39.6° corresponding to metallic Pt for PCO and PZCO was observed verifying previous observations from Pilger et al.^[^ ^62^ ^]^ who observed this segregation effect under oxidizing conditions at high temperatures. This also confirms the successful incorporation of Pt within the Gd‐doped CeO_2_ matrix making this sample more promising when it comes to potential stability issues under reaction streams.

Peak shifting toward higher 2*θ* values was observed when Zr^4+^ was incorporated within the fluorite structure, while this shift did not occur when Gd^3+^ was doped. This is because Ce^4+^ and Gd^3+^ have similar sizes (0.97 and 1.05 Å, respectively). Conversely, the substantial 15% difference in ionic radii between Ce^4+^ and Zr^4+^ (0.97 and 0.84 Å, respectively) reduces the unit cell dimensions and consequently the interplanar spacing between cells (*d*) which is ultimately inversely related to the 2*θ* values, according to Bragg's Law, resulting in a noticeable peak shift toward higher angles.^[^ ^63^ ^]^

Broadening of the peaks for PCZO in the X‐ray patterns illustrated in Figure 2a was also observed. For PCZO, a lower crystal size was observed as compared to its analogues, which was previously observed when Zr was doped in CeO_2_ matrices.^[^ ^64^ ^]^ This was further confirmed by the calculated average crystal size values: 4.9 ± 0.8, 2.6 ± 0.6, and 4.2 ± 0.5 nm for PCO, PCZO, and PGCO, respectively. The microstructure of the material also seems to be affected by Zr‐doping, as less defects and pores were observed for PCZO according to the SEM analysis (Figure 2b and S7, Supporting Information). On the other hand, it is also evident that the Gd‐doping maintained a similar microstructure as compared to the parent PCO. Nevertheless, their total surface area remains similar and quite low at ≈2–4 m^2^ g^−1^.

### 2.3 Inducing Pt Exsolution from Mixed Simple Oxides

**Figure 3:**
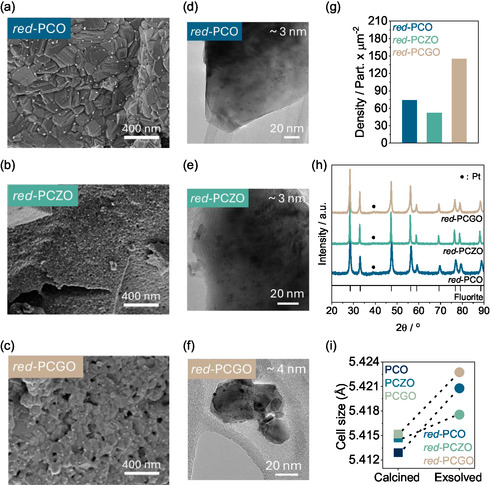
PCO and doped PCO materials after undergoing reduction at 900 °C for 10 h. a) Red‐PCO; b) red‐PCZO; c) red‐PCZO; and d–f) TEM images of red‐PCO, red‐PCZO, and red‐PCGO, respectively. g) Population density of exsolved nanoparticles. h) PXRD of the three different species. Exsolved Pt appears indicated as •. i) Lattice parameters comparison of the as sintered and reduced samples.

The as‐synthesized materials described above were reduced under 100% H_2_ at 900 °C for 2 h to induce Pt exsolution. As observed in the SEM images displayed in **Figure** **3**a–c, emergence of Pt nanoparticles was achieved in all cases. XRD spectra (Figure 3h) also confirmed the formation of metallic Pt, visible from the increase of a peak at 39.5°, corresponding with the main Pt^0^ peak. The slight asymmetry observed solely for the PCGO pattern could be attributed to the presence of a small contribution of another fluorite phase (tetragonal, orthorhombic, etc.) or crystallographic effects such as strain in the crystal lattice, which may lead to distortions, ultimately manifested as the shoulder observed in the pattern. Refinement revealed that amount of Pt exsolved on the surface was of the order of magnitude of 25% of the originally doped for all materials.^[^ ^55^ ^]^ Concurrently, the materials successfully maintained their main CeO_2_ structure. No additional peaks were observed in the PXRD spectra, indicating that no secondary phases were formed upon reduction beyond the metallic Pt.^[^ ^50^ ^]^ Rietveld refinement of the spectra relative to the calcined and exsolved materials was conducted for further confirmation of the reduction of the materials. As phase identification of the fluorite and platinum phases was previously done, the main interest lays on additional parameters of the fluorite and its evolution before and after reduction, such as lattice parameters. Refinement revealed that all the materials underwent cell expansion after the reduction (Figure 3i), as a consequence of the transformation of Ce^4+^ into Ce^3+^. Quality and accuracy of the refinements were assessed by the coefficients displayed: 8.2, 4.2, and 7.0% for red‐PCO, red‐PCZO and red‐PCGO, respectively.

Figure 3a–f and S7 (Supporting Information) show the SEM and TEM images of the different materials after exsolution. Pt particles with different size and distribution can be observed in all the materials. Unlike red‐PCZO, red‐PCO and red‐PCGO adopted a similar structure with homogeneous exsolution to the previously reported by Carrillo et al.^[^ ^37^ ^]^ However, it is interesting to note that after reduction, the Zr and Gd doped samples show porous microstructure, with larger pores for the Gd‐doped than for the Zr‐doped. Also, red‐PCZO fewer exsolved particles seem to be formed on the surface as compared to the other two, which could be explained by the difference in the microstructure where bigger matrix crystal size would hinder the long diffusion of ions towards the surface and hence result in overall lower extent of exsolution. This is further confirmed by the population density measurements (Figure 3g), conducted with ImageJ as described in the methods. red‐PCO had a population density of ≈74 particles per μm^2^, while red‐PCZO had the lowest density, with 52 particles per μm^2^. In contrast, red‐PCGO exhibited a substantial increase in the amount of exsolved particles, reaching 145 particles per μm^2^, doubling the quantity of particles exsolved in the other samples. The average particle size has been measured both by SEM and TEM. However, TEM measurements are usually more precise when it comes to small nanoparticles as the resolution of SEM could lead to overestimation of the size. Nevertheless, SEM allows us to look into the distribution of the particles’ sizes relative to the samples better rather than the absolute values (Figure S10, Supporting Information). The average particle size of the exsolved Pt metallic nanoparticles measured by TEM is displayed in Figure S9 (Supporting Information). In terms of Pt particle size, the average size measured by TEM in red‐PCO and red‐PCZO was ≈3 nm, while red‐PCGO showed an average size of ≈4 nm. This implies that overall, the Gd‐doped sample has allowed for more Pt ions to migrate to the surface and exsolve as expected due to the higher defects in the PCGO structure, resulting from the varying oxidation states of Gd and Ce and the smaller crystal size of the matrix.

**Figure 4:**
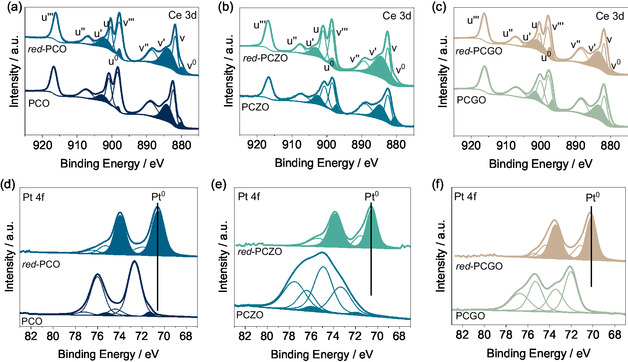
Ce 3*d* XPS spectra of a) PCO, b) PCZO, and c) PCGO. Colored curves correspond to Ce^3+^. Pt 4*f* spectra of d) PCO, e) PCZO, and f) PCGO. Colored curves correspond to Pt^4+^, when present. Each image is depicting two different spectra. Bottom ones correspond to as sintered materials. Top ones correspond to the materials after undergoing reduction to promote Pt exsolution.

The surface of the materials before and after emergence of the Pt phase was also studied by XPS. **Figure** **4**a–f shows the different spectra for Ce and Pt of the three different catalysts before and after reduction. Correction of the binding energy (BE) was done by alignment of the C 1*s* peak. The presence of tetravalent Ce^4+^ was confirmed for all the three catalysts by the characteristic *u‴* component, at 916 eV, in the higher BE (see Figure 4a–c).^[^ ^65^ ^]^ Peaks at 882, 888, 898, 900, and 907 eV were identified, indicating a mixture between Ce 3*d* ^9^ 4*f* ^2^ O 2*p* ^4^ and Ce 3*d* ^9^ 4*f* ^1^ O 2*p* ^5^.^[^ ^66^, ^67^ ^]^ Further Ce 3*d* spectra deconvolution confirmed the existence of Ce (III) species in all the materials before and after exsolution. Peaks at 886 and 902 eV (namely *u*′ and *v*′) corresponded to the Ce 3*d* ^9^ 4*f* ^1^ O 2*p* ^6^ final state.^[^ ^67^ ^]^ Peaks at 881 and 898 eV (*v* ^0^ and *u* ^0^, respectively) were also ascribed to Ce^3+^ species.^[^ ^50^, ^67^ ^]^

XPS spectra of PCO, PCZO, and PCGO for Pt 4*f* before and after exsolution are depicted in Figure 4d–f. Two sets of spin‐orbit coupling were identified, Pt 4*f*_5/2_ at higher BE, and 4*f*_7/2_ at lower BE. Peaks at lower BE in each multiplet corresponds to metallic Pt, while peaks at higher BE correspond to electron deficient platinum species (i.e., Pt^2+^ and Pt^4+^).^[^ ^68^ ^]^ Before reduction, the peaks of PCO and PCZO could be fitted with three curves in both Pt 4*f*_5/2_ and Pt 4*f*_7/2_ (Figure 4d,e, bottom curves). This was in accordance with our XRD data that revealed the presence of a small amount of Pt^0^ in the two sintered samples. On the other hand, metallic Pt was not detected for PCGO (Figure 4f, bottom curves) corroborating with the PXRD results, where no‐segregation of Pt was observed.

Pt^0^ peaks shifted from 71.4 to 70.5 eV and from 71.8 to 70.5 eV for red‐PCO and red‐PCZO, respectively while for the red‐PCGO, Pt^0^ peak appeared at 70.2 eV. Peaks shifting to lower BE upon reduction is a consequence of the formation of larger content of Pt in a lower oxidation state (Pt^0^),^[^ ^68^, ^69^ ^]^ also confirming the exsolution of Pt nanoparticles.

The quantification of the ratio between Ce^3+^ and Ce^4+^ in our materials could help determine qualitatively the formation of oxygen vacancies, as the reduction of Ce^4+^ to Ce^3+^ is translated into a lower oxygen content within the material. This was also previously implied from refinement of the XRD spectra and the study of lattice expansion, which is directly related to the formation of vacancies.^[^ ^70^, ^71^ ^]^ The number of oxygen vacancies increased across the board, as expected, after reduction for all the materials. The O 1*s* XPS of all samples (Figure S2, Supporting Information) was fitted to two main peaks, one at 531.5 eV and another at 529.5 eV. The former corresponds to adsorbed species (such as O^−^, O_2_^−^, OH^−^, or contaminants), and the latter corresponds to lattice oxygen (O^2−^). No significant changes were observed for the lattice oxygen, while a decrease in the peak corresponding to the adsorbed species was noticed for all the three materials upon reduction, indicating the formation of oxygen vacancies,^[^ ^72^ ^]^ thus supporting our hypothesis.

When comparing the effect of doping Zr and Gd in the CeO_2_ support matrix (**Table** **1**), it could be observed that Zr‐doped materials demonstrate a larger Ce^3+^:Ce^4+^ ratio (0.45 and 0.23 for PCZO and PCO, respectively). As the cationic radius of Zr^4+^ is significantly smaller than Ce^4+^, distortions are created in the fluorite structure, thus enhancing the reducibility of the material and eventually creating more oxygen vacancies and expecting to decrease the coordination number of the Zr^4+^ cations.^[^ ^73^ ^]^ This decrease in coordination number could indeed be correlated to the creation of more oxygen vacancies in PCZO also according to previous simulation^[^ ^74^ ^]^ and experimental^[^ ^75^ ^]^ published literature. As discussed previously, upon reduction, the amount of oxygen vacancies was expected to increase. This number increased even more, reaching 0.55 for red‐PCZO sample, indicating an increase in the amount of these vacancies upon exsolution, creating more oxygen vacancies than its undoped reduced analogue, red‐PCO, with a Ce^3+^:Ce^4+^ ratio of 0.49. On the other hand, it is expected that Gd‐doped CeO_2_ would have more oxygen vacancies than the undoped CeO_2_, due to charge compensation, as Gd is a trivalent cation substituting the mostly tetravalent Ce cations. PCGO displayed an initial Ce^3+^:Ce^4+^ ratio 0.34, while the value was of 0.23 for PCO. After reduction, the same trend was maintained as red*‐*PCGO increased its Ce^3+^:Ce^4+^ ratio up to 0.59, while the value for red‐PCO increased up to 0.49. This means that Zr‐ and Gd‐doping indeed generated larger amount of surface oxygen vacancies in both the as sintered materials and upon reduction. This result also agrees with previous observations.^[^ ^76^, ^77^ ^]^ However, overall, red‐PCGO combined the largest amount of oxygen vacancies among the three materials, with the enhanced microstructural properties, such as larger porosity and the formation of optimal amount of exsolved nanoparticles. Therefore, this could be an indication that these materials could be the best performing and/or stable one for our intended application.

**Table 1:** Weight fraction on the materials’ surface. Species represented: Ce^3+^, Ce^4+^, and ratio between Ce^3+^:Ce^4+^. The later has been calculated as qualitative approach to the formation of surface oxygen vacancies.

Catalyst	Ce^3+^ [wt%]	Ce^4+^ [wt%]	Ce^3+^:Ce^4+^
PCO	21.60	78.40	0.23
Red‐PCO	33.06	66.94	0.49
PCZO	31.12	37.41	0.45
Red‐PCZO	35.60	64.40	0.55
PCGO	25.19	74.81	0.34
Red‐PCGO	37.22	62.78	0.59

The Raman spectra of all samples before and after reduction are presented in Figure S3a,b (Supporting Information). A strong peak observed at 460 cm^−1^ is attributed to the F_2g_ mode of the *Fm‐3m* fluorite structure of CeO_2_. This mode corresponds to the symmetrical stretching vibrations of oxygen atoms surrounding Ce^4^^+^ ions (Ce–O).^[^ ^78^ ^]^

In addition to the F_2g_ mode, the unreduced and reduced samples display a broad band in the 550–600 cm^−1^ range, referred to as the “D band,” which is associated with structural deformations, defects, and oxygen vacancies.^[^ ^79^ ^]^ This D band can be deconvoluted into two components, D1 and D2, for unreduced samples as shown in Figure S3 (Supporting Information), with peaks centered around 560 and 590 cm^−1^, respectively. The D1 band is attributed to extrinsic oxygen vacancies created to maintain charge neutrality when Ce^4^^+^ ions are substituted by trivalent cations (e.g., Gd^3^^+^). In contrast, the D2 band, observed at ≈590 cm^−1^, is linked to intrinsic oxygen vacancies associated with the presence of Ce^3^^+^ ions.^[^ ^80^ ^]^

However, after reduction, as shown in the Figure S3d (Supporting Information), the intensity of the D band component near 560 cm^−1^ increases relative to the component at 590 cm^−1^, indicating an increase in the oxygen vacancies following H_2_ reduction. Previous studies^[^ ^81^, ^82^, ^83^ ^]^ have associated the peak near 560 cm^−1^ with Raman‐active defects located in the surface layer and generated during H_2_ reduction in ceria, whereas the peak at 590 cm^−1^ refers to inner vacancies and are not affected by reduction at all. Additionally, a broad band around 700 cm^−1^ is observed in the unreduced samples, which is attributed to the symmetric stretching vibrations of bridging Pt—O—Ce bonds. Notably, this band completely disappears after reduction, indicating the exsolution of Pt metal onto the surface from the host lattice.^[^ ^84^ ^]^

The larger exsolution extent in Gd‐doped CeO_2_ is linked to the higher oxygen vacancy concentration introduced by trivalent Gd^3^^+^ dopants, which facilitates Pt migration, promotes nucleation, and enhances nanoparticle stability, as postulated by Weber et al.^[^ ^85^ ^]^ They demonstrated that acceptor doping (such as Gd^3+^) increases oxygen vacancies, promoting faster nucleation and reducing nanoparticle coalescence, aligning with our findings.

### 2.4 Biosensing Properties of MFCs with Exsolved Mixed Oxides Catalysts for ORR

After developing and characterizing red‐PCGO, red‐PCO, and red‐PCZO, these materials were tested as catalysts in the air‐cathode of MFC biosensors. The tests aimed to assess their effectiveness in catalyzing ORR and their impact on the biosensor's ability to detect organic pollutants in urban artificial wastewater (AWW).

**Figure 5:**
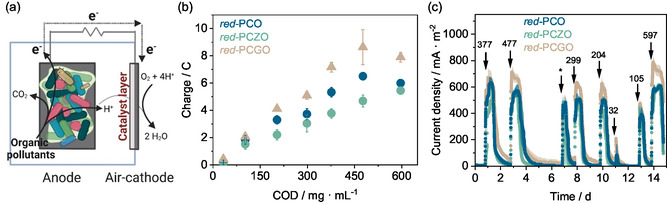
a) Schematic representation of an MFC‐based biosensor. b) Chronomaperogram of the MFC‐based biosensors with different catalysts for ORR: red‐PCGO, red‐PCO, and red‐PCZO. The biosensors were fed in batch mode with AWW. The COD concentration of AWW varied at each batch, resulting in different current peaks. c) Calibration curve of MFC‐based biosensors with the three different catalysts tested. The calibration curves show the correlation between the charge produced versus the COD of the AWW inserted in the biosensors. Data are presented as the averages of two MFC replicates for each catalys*t* tested ± standard error of the mean (*n* = 2) *X*‐axis standard deviation bars are not visible as they are smaller than the symbols. The asterisk (*) respresents a peak where the data logger did not work properly due to a fault, so it was discarded.

AWW with varying chemical oxygen demand (COD) concentrations (from 32 to 597 mg L^−1^ COD) but stable conductivity (1.22 mS cm^−1^) was introduced into the biosensor in batches. A schematic representation of the biosensor is shown in **Figure** **5**a. The current response was monitored to determine if a correlation existed between the electrical response and the COD concentration. As shown in Figure 5c, a current peak is observed at each batch when the AWW is completely replaced with fresh AWW in the biosensor. This peak is attributed to the electroactive bacteria at the anode, which starts metabolizing the substrate present in the AWW, transferring electrons to the anode and causing an increase in current. Once the biofilm depletes the oxidizable substrates, the current decreases as no more electrons are transferred to the anode.^[^ ^86^ ^]^ Increasing the COD of the AWW results in a higher charge generation by the MFCs, as the biofilm has more substrate to oxidize. This demonstrates the MFC's potential as a biosensor for total oxidizable contaminants in wastewater, operating without the need for manual intervention or external power supply.^[^ ^87^ ^]^

The biosensing properties are influenced by the MFC's capability to generate current and, consequently, charge (calculated as the integral of current over time, as shown in Equation (2)). The maximum current density (current normalized by the anode's projected area of 12 cm^2^) achieved by the MFCs at room temperature (COD = 597 mg L^−1^, conductivity = 1.24 mS cm^−1^, pH = 7.38, external load of 100 Ω) was highest for MFCs with red‐PCGO catalyst (788 ± 46 mA m^−2^), followed by red‐PCO (633 ± 41 mA m^−2^) and red‐PCZO (621 ± 15 mA m^−2^). This indicates superior electrochemical performance and current response for red‐PCGO. Additional electrochemical analyses were conducted to validate these findings and are detailed in the experimental section (COD biosensing).

The linear regressions established for the correlation between COD and the charge generated by MFCs with different catalysts were *y* = 0.018*x* + 0.057 for red‐PCGO, *y* = 0.010*x* + 0.148 for red‐PCZO, and *y* = 0.014*x* + 0.130 for red‐PCO. The corresponding coefficients of determination (*R* ^2^) were 0.95, 0.91, and 0.95, respectively, indicating a strong correlation between the electrical response of all biosensors and the COD of the wastewater.

Sensitivity refers to the magnitude of change in charge produced by the biosensor in response to variations in COD concentration, and it is represented by the slope of the calibration curves. A more sensitive biosensor can more accurately detect small changes in COD.^[^ ^88^ ^]^ Among the MFCs with the three catalysts tested, the most sensitive was the one with the red‐PCGO catalyst, exhibiting a sensitivity of 0.018 ± 0.002 C mg^−1^ L^−1^, which was slightly lower than the conventional Pt/C catalyst at 0.021 ± 0.002 C mg^−1^ L^−1^, as shown in Figure S6 (Supporting Information). The next most sensitive biosensor was the one with the red‐PCO catalyst (0.014 ± 0.002 C mg^−1^ L^−1^), followed by the red‐PCZO catalyst (0.010 ± 0.001 C mg^−1^ L^−1^). The sensitivity of the biosensor is determined by its ability to generate a strong electrical response to small changes in COD. The higher sensitivity of MFCs with the red‐PCGO catalyst indicates superior current generation in the MFC compared to red‐PCO and red‐PCZO. This higher value is attributed to the combination of the creation of a high number of oxygen vacancies,^[^ ^80^ ^]^ the stability of the newly formed material, and the higher exsolution particle density of red‐PCGO, which was around 2 and 3 times higher than the amount of red‐PCO and red‐PCZO, respectively.

Limit of detection (LOD) is defined as the lowest concentration of an analyte that the biosensor can reliably detect (but not necessarily quantify with accuracy).^[^ ^89^ ^]^ The LOD values for the three biosensors are 134 ± 11 mg L^−1^ for red‐PCGO, 133 ± 11 mg L^−1^ for red‐PCO, and 196 ± 24 mg L^−1^ for red‐PCZO. The maximum linear detection range was similar for red‐PCGO and red‐PCO, both up to 477 mg L^−1^, while for red‐PCZO, it extended to 597 mg L^−1^. The maximum detectable COD concentration is constrained by the fact that beyond this concentration, any further increase in COD does not result in a corresponding increase in charge. This is attributed to the slower oxidation rate of excess substrate by electroactive bacteria, which allows competitive reactions to occur. These may involve non‐electroactive bacteria competing for the substrate, such as fermentation in the absence of oxygen or aerobic metabolism when oxygen is available. The latter is more likely, as the biosensor was not maintained under anaerobic conditions, and oxygen leakage through an air cathode is a well‐documented issue in the literature.^[^ ^89^, ^90^ ^]^

The response time, defined as the time interval required to generate each peak (using a current baseline of 25 mA cm^−2^), increases with the rise in COD for all types of biosensors, consistent with previously observed.^[^ ^91^ ^]^ In MFCs, the response time did not vary significantly with the catalyst used, ranging from 21.5 ± 0.1 to 73.6 ± 2.6 h for red‐PCGO, 17.8 ± 0.3 to 75.3 ± 1.8 h for red‐PCO, and 13.9 ± 0.6 to 65.3 ± 1.4 h for red‐PCZO. These values are comparable to those obtained with the conventional Pt/C catalyst, where the response time ranged from 13.4 ± 1.2 to 62.3 ± 1.0 h. The response times observed in this study are also consistent to those reported in other studies using MFC‐based biosensors with similar system configurations and calibration approaches.^[^ ^91^, ^92^ ^]^ The proposed method allows for the determination of COD/BOD in a significantly shorter time compared to the 5‐day period required by the standard ISO method while also eliminating the need for manual intervention and external power supply.^[^ ^93^ ^]^ However, response time can be significantly reduced to the order of minutes, especially when the system is miniaturized and operated in continuous flow under optimized flow rate conditions.^[^ ^94^ ^]^

Polarization tests were conducted at the start of the experiment when the electroactive biofilm on the anode was fully developed and producing a stable current signal (after 4 weeks from the inoculum), and the air cathode was newly installed in the biosensors. The tests were repeated after 14 days of biosensor operation with AWW to assess any changes in the electrochemical performance of the MFCs, with a particular focus on the cathode and overall cell performance, as air cathodes are frequently identified as a limiting factor for maintaining current stability over time.^[^ ^95^, ^96^, ^97^ ^]^

**Figure 6:**
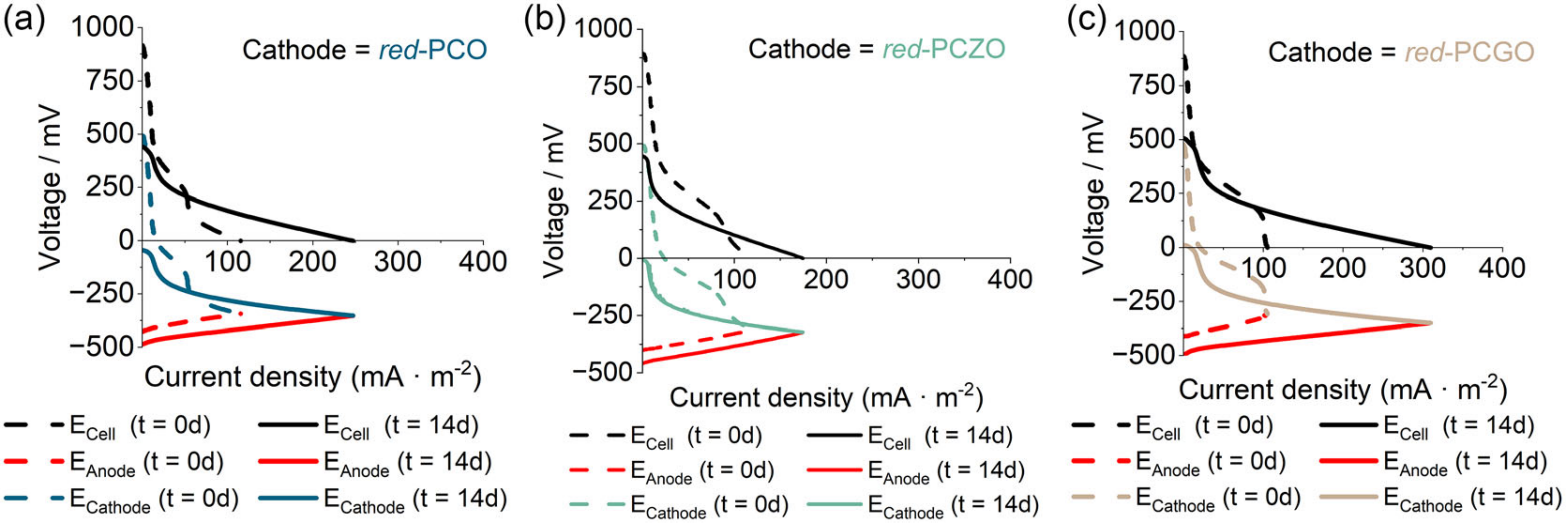
Polarization curves for the cell, anode, and cathode of MFCs equipped with a) red‐PCO, b) red‐PCZO, and c) red‐PCGO as catalyst for ORR on the air cathode. Polarization measurements were conducted at the beginning of the experiment with a new cathode (dotted line) and after 14 days of continuous biosensor operation (solid line).

At the start of the experiment (*t* = 0 d), all three types of biosensors achieved comparable maximum current densities, as illustrated in **Figure** **6**a–c, with red‐PCZO exhibiting the highest value at 324 mA m^−2^, followed by red‐PCO at 310 mA m^−2^ and red‐PCGO at 284 mA m^−2^. Although differences were observed, they were not statistically significant. The open circuit potential (OCP) of the MFCs was measured at 895 mV for red‐PCZO, 918 mV for red‐PCO, and 889 mV for red‐PCGO. Correspondingly, the cathode OCP values were 489 mV for red‐PCZO, 490 mV for red‐PCO, and 477 mV for red‐PCGO, while the anode OCPs were −399, −424, and −411 mV, respectively. These values are indicative of a well‐developed electroactive biofilm on a carbon electrode, consistent with typical electrochemical behavior observed under standard operating conditions.^[^ ^98^ ^]^

The polarization curves of the cells show a sharp voltage drop at low current densities for all three catalysts, indicating that activation losses are the primary contributors to the internal resistance of the MFCs.^[^ ^86^ ^]^ This effect is primarily attributed to the cathode, as evident from the cathode polarization curves (cathodic dotted lines in Figure 6a–c), which exhibit a steep decline in voltage at low current densities. In contrast, the anode polarization curves (black dotted lines in Figure 6a–c) do not show this significant drop, confirming that the cathode is the main source of activation losses across all three catalysts.

After 14 days of operating the biosensor with AWW, polarization tests were repeated. The maximum current density of the biosensor increased for all three types of catalysts compared to the initial measurements, reaching 843 mA m^−2^ for red‐PCGO, 667 mA m^−2^ for red‐PCO, and 470 mA m^−2^ for red‐PCZO. Among the catalysts tested, red‐PCGO exhibits the highest performance in terms of current production, and the result is consistent with the biosensing properties discussed previously in this section, where MFCs with red‐PCGO demonstrated superior performance in terms of charge generation, sensitivity, and limit of detection, followed by red‐PCO and red‐PCZO.

We attribute this significant difference to the exsolution of Pt nanoparticles. Red‐PCGO preserved a homogeneous distribution of particles and larger number of nanoparticles upon reduction. Furthermore, due to its enhanced conductivity and stability during these processes, Gd‐doped ceria tends to be a common material used in fuel cell research.^[^ ^16^ ^]^ The highest maximum current density obtained at *t* = 14 d compared to *t* = 0 d is primarily due to increased biological activity at the anode, as evidenced by a further reduction in the anode OCP to −495 mV for red‐PCGO, −486 mV for red‐PCO, and −444 mV for red‐PCZO. The cathode OCP dropped significantly for all three catalysts, reaching 13 mV for red‐PCGO, −43 mV for red‐PCO, and −12 mV for red‐PCZO. Consequently, the overall cell OCP decreased to 508, 443, and 450 mV for red‐PCGO, red‐PCO, and red‐PCZO, respectively. However, the steep voltage drop at low current densities was no longer observed at *t* = 14 days, and the electrochemical behavior at high current densities (and low external loads) remained stable for all three catalysts. This stabilization, along with the enhanced biological activity at the anode throughout the experiment, contributed to the sustained increase in current density over time in MFCs with all three catalysts.

MFC with red‐PCGO catalyst exhibited electrochemical properties comparable to those of the conventional Pt/C catalyst, which reached a maximum current density of 349 mA m^−2^, at *t* = 14 days, with a cell OCP of 575 mV (Figure S6b, Supporting Information). We attribute this enhanced performance to the higher extent of Pt nanoparticles and enhanced stability of the catalyst. However, while the cathode OCP in the Pt/C catalyst significantly declined and the cell voltage remained lower across all current densities (and external loads), the exsolved catalysts presented here displayed a decrease in cell OCP without substantial changes in cathode performance at higher current densities compared to *t* = 0 days. This indicates that exsolved Pt catalysts may offer better operational stability than Pt/C for biosensing applications, which is prone to degradation due to Pt particle agglomeration, detachment, poisoning, and fouling.^[^ ^99^, ^100^, ^101^ ^]^ This hypothesis is further supported by cyclic voltammetry tests conducted at *t* = 0 and *t* = 14 days (Figure S6c, Supporting Information). These cyclic voltammetries showed minimal changes in the voltammogram profiles for the exsolved catalysts, whereas the Pt/C catalyst exhibited a significant reduction in the voltammogram area, indicating a decrease in the electrochemically active surface area of the catalyst.^[^ ^102^ ^]^ This further highlights the potential of the exsolved samples presented here as long‐term stable, efficient catalysts. Unfortunately, due to the formation of a biological layer on the cathode surface during the operation of MFC‐based biosensors (Figure S8, Supporting Information), it was not possible to characterize these samples to further enhance this hypothesis.

## 3 Conclusions

In this study, we employ the exsolution method to study exsolution of Pt systems from Zr, Gd, and non‐doped CeO_2_. The results presented here show controlled emergence of metallic Pt nanoparticles exsolved from CeO_2_‐based matrices. Their effect on and in conjunction with exsolution has never been studied before. We also demonstrate that doping the CeO_2_ matrix with either Zr or Gd results in an increase in oxygen vacancies upon reduction when promoting the exsolution of these Pt nanoparticles. Our findings show that Gd‐doped CeO_2_ successfully achieves a larger density and more homogeneous distribution of Pt nanoparticles as compared to its undoped and Zr‐doped CeO_2_ analogues. Additionally, this sample also exhibits the highest performance, achieving superior sensitivity and the lowest limit of detection, comparable to that of conventional Pt/C catalysts. This enhanced performance is attributed to the combination of increased oxygen vacancies and a greater amount of exsolved nanoparticles. Polarization tests revealed that, although there was a decrease in the initial cathode open‐circuit potential, the cathode voltage remained stable at high current densities (when comparing the polarization curves at *t* = 0 d and *t* = 14 d), indicating enhanced operational stability of the electrode under closed circuit conditions. In contrast, the Pt/C catalyst demonstrated a consistent decline in cathode voltage across all current densities. These findings pave the way for the development of advanced yet simple material designs for catalytic applications. The approach outlined in this research holds significant potential for impacting other fields in catalysis, given the versatility and effectiveness of such catalysts in a wide range of processes.

## 4 Experimental Section

4.1

4.1.1

##### Materials Synthesis

Cerium oxide (CeO_2_) was prepared by calcination of cerium (III) nitrate hexahydrate (99.99%, Sigma‐Aldrich) in static air at 600 °C for 6 h (CeO_2_‐1). All other catalysts used in this study, CeO_2_‐2, PCO, PCZO, and PCGO, were prepared by modified Pechini method.^[^ ^37^ ^]^ Here, the stoichiometric notation represented the atomic ratios of the elements, while the platinum loading was ≈2 wt% in all the Pt‐containing catalysts. Required amounts of cerium (III) nitrate hexahydrate (99.99%, Sigma‐Aldrich), platinum (IV) nitrate solution (Pt 15 wt%, Fisher Scientific), zirconium dinitrate oxide hydrate (99.99%, Sigma‐Aldrich), and gadolinium(III) nitrate hexahydrate (99.99%, Sigma Aldrich) were dissolved in aqueous solution of citric acid (CA, metal precursor/CA molar ratio of 2/3, 99.5%, Sigma‐Aldrich) at 60 °C under constant stirring for 3 h. Subsequently, ethylene glycol (EG; mass ratio EG/CA = 3/2 wt%; 99.8%, Sigma‐Aldrich) was added, and the temperature was set to be increased to 80 °C and stirred for 2 h until the solvent was evaporated and became foamy solution. The solution was then dried overnight in an oven at 120 °C and calcined at 700 °C under air at atmospheric pressure for 16 h to obtain the desired materials (CeO_2_‐2 and the respective PCO, PCZO, and PCGO). The resulting powders were collected and grounded to a fine powder with a mortar and pestle.

Samples were loaded in a horizontal tubular furnace at 900 °C for 2 h under 100% H_2_ to promote nanoparticle exsolution. The samples were named PCO, PCZO, and PCGO after calcination, and red‐PCO, red‐PCZO, and red‐PCGO denoted the samples after reduction, respectively.

##### Material Characterization

The crystalline structures of all samples were analyzed with the PXRD method (X’Pert Powder from PANalytical; Cu–Kα radiation; *λ* = 0.15406 nm), operated at 40 kV and 30 mA. The diffraction patterns were collected over a 2*θ* range from 10° to 90° at a scanning rate of 0.011° s^−1^. The XRD patterns were collected both before and after the exsolution process. Pt and crystallite sizes were calculated using Debye–Scherrer (Equation (1)).^[^ ^103^ ^]^

(1) $$D=\frac{K\lambda }{\beta \text{cos}\left(\theta \right)}$$

where *D* is the average particle diameter (nm), *K* is the dimensionless shape factor (assumed to be 0.94 in this study), *λ* is the wavelength of the X‐ray radiation used, *β* is the full width at half maximum (FWHM) of the diffraction peak in radians, and *θ* is the diffraction angle in degrees.

SEM images of both before and after the reduction of the samples were obtained by Thermo Fischer Apreo2 SEM operating at 2 kV and 0.1 nA. The SEM images were taken with a magnification range of 1000–100 000 at a working distance of 3 mm. ImageJ was used to investigate the platinum particle size and population density on the catalyst surface.

XPS analyses were performed on a Thermo Fisher Scientific (East Grinstead, UK) K‐Alpha+ spectrometer. XPS spectra were acquired using a monochromated Al Kα X‐ray source (*hv* = 1486.6 eV). An X‐ray spot of ≈400 μm radius was employed. Survey spectra were acquired employing a pass energy of 200 eV. High‐resolution, core‐level spectra for all elements were acquired with a pass energy of 50 eV. All high‐resolution core level spectra were charge referenced against the C 1*s* peak at 284.8 eV to correct for charging effects during acquisition. Quantitative surface chemical analyses were calculated from the high‐resolution, core‐level spectra following the removal of a nonlinear (Shirley) background for Ce spectra and a linear background for Pt spectra. Spectrum of the Ce 3*d* level provided two multiplets, *u* (higher BE) and *v* (lower BE). *u* and *v* labels indicated the spin‐orbit coupling 3*d*_3/2_ and 3*d*_5/2_, respectively.^[^ ^66^ ^]^ CasaXPS software was used for XPS analysis, which incorporated the appropriate sensitivity factors and corrected the electron energy analyzer transmission function.

Raman spectra of both fresh and reduced samples were obtained using a Renishaw InVia Reflex Raman Microscope (Renishaw, UK) with a 532 nm laser as the excitation source. Spectral deconvolution (band analysis) was performed using OriginLab's Origin software.

TEM analysis was performed on reduced samples using the Talos F200I instrument from Thermo Fisher, equipped with a 200 V electron source. For sample preparation, the powder was dispersed in ethanol using an ultrasonic bath and then deposited onto copper grids coated with a lacey carbon film and dried.

##### MFC Assembly and Catalyst Ink Preparation: Air‐Cathode Preparation

Gas diffusion cathodes were made with carbon cloth electrodes (W1S1011, FuelCell Store, Texas, USA). A catalyst loading of 0.06 mg cm^−2^ was used on the cathode's surface. The appropriate % of Pt loading was chosen after optimization that had been addressed in a separate study and was out of scope here. The exsolved catalysts were tested in duplicates and compared with Pt/C with the same catalyst loading. Nafion was used as binder for applying catalyst layer on the cathodes, as reported in previous studies.^[^ ^104^, ^105^ ^]^ Isopropanol was added into only Pt/C to increase the solubility when constructing Pt/C cathode. The cathodes were dried at room temperature overnight. Cathodes were named as Pt/C, red‐PCO, red‐PCZO, and red‐PCGO. After preparing the air cathode, it was assembled into the MFC with the catalyst layer oriented toward the electrolyte.

##### MFC Assembly and Catalyst Ink Preparation: MFC Setup and Operation

Eight single chambers MFCs with an air‐breathing cathode were built. A cube (5 × 5 × 5 cm) with a cylindrical chamber in the middle of 30 mL and two lids to close the ends of the reactor, one filled and the other with a circular hole (3 cm diameter) in the center for the air‐cathode side. The reactor was assembled by adding custom‐made silicon gaskets in between the plates and the main body of the reactor to avoid leakage. The external chassis of the reactor was made of Co‐Polyester+ (Ultimaker, Utrecht, NL) and built with a 3D printer (Ultimaker 3, Ultimaker B.V, Utrecht, NL). Anode was prepared using a 3 cm × 2 cm piece of carbon felt (6.35 mm thick, 99.0%, Alfa Aesar, Lancashire, UK) connected to a titanium wire (the Crazy Wire Company, UK), which was used to connect the circuit to an external load of 100 Ω. MFCs were fed with different concentrations of AWW which was prepared as previously described^[^ ^106^ ^]^ in a stock of 10× concentration. The stock was sterilized by autoclaving at 121 °C for 15 min. The concentrated AWW was diluted with different rations of DI water and phosphate buffer saline to obtain different BOD concentrations while maintaining similar conductivity (1.2 ± 0.2 mS cm^−1^) and pH (7–7.5). Considering that in AWW the ratio between BOD and COD remains stable, COD was measured routinely as it provides results in a shorter timeframe (a few hours) compared to BOD, which requires several days for accurate determination.^[^ ^87^ ^]^ The BOD/COD ratio of the AWW used in the study was 0.51.^[^ ^95^ ^]^ COD was measured using the standard method ISO 6060‐1989 using a COD cuvette test (Hach Lange GmbH, Germany).

The biofilm around the anode was developed from an inoculum sourced from an acetate‐fed MFC. The inoculum was centrifuged for 10 min at 4000 rpm and resuspended in a solution of 1.5× concentrated AWW (COD = 596.5 mg L^−1^, conductivity = 1.22 mS cm^−1^, pH = 7.42). Optical density (OD_600_) was determined to be 0.5, found with a spectrophotometer (Ultrospec 2000, Pendragon Scientific Ltd, UK) at 600 nm wavelength. 10% v/v inoculum in 1.5× concentrated AWW (COD = 596.5 mg L^−1^, conductivity = 1.22 mS cm^−1^, pH = 7.42) was used to inoculate the MFCs. This was emptied and refilled when the substrates within it were completely oxidized (around 1–4 days). Once the MFCs maximum current output remained stable (variation of ±10 μA) for three AWW replacements at same COD, the biofilm was considered fully developed.

##### Electrochemical Analysis

Electrode and cell voltages were measured in all reactors using a PicoLog data logger (PicoTech, UK) after replacing the electrolyte and leaving the MFCs in OCP for 3 h. Polarization studies were carried out via linear sweep voltammetry (LSV) using a potentiostats (Palmsens4 and Emstat 2, Palmsens, NL) at a scan rate of 0.5 mV s^−1^ from cell OCP to 0 V, connected in two‐electrode mode (i.e., anode as working electrode and cathode as both counter and reference electrode). 1.5× concentrated AWW (COD = 596.5 mg L^−1^, conductivity = 1.22 mS cm^−1^, pH = 7.42) was used as electrolyte. Current density, power density, and internal resistance were calculated as previously reported.^[^ ^86^ ^]^ Individual electrode currents were also measured during the LSV. Cyclic voltammograms of the cathode were obtained using the same AWW as electrolyte. Cyclic voltammetry (CV) was carried out in three‐electrode mode with the cathode as the working electrode, an Ag/AgCl (3 m KCl) as reference electrode, and a Pt wire as counter electrode. A scan rate of 5 mV s^−1^ was used within a potential range of −1.0–1.0 V. Both LSV and CV studies used 1.5× concentrated AWW solution (COD = 596.5 mg L^−1^, conductivity = 1.22 mS cm^−1^, pH = 7.42). The electrochemical analyses were performed at the beginning of the experiment and repeated after 14 days, to evaluate changes in the electrodes’ performance.

##### COD Biosensing Properties

The charge generated by the MFC devices with different catalysts for ORR (two MFC replicates for each catalys*t* tested) was calculated with Equation (2):

(2) $$Q=\int_{t_{1}}^{t_{2}}Idt$$

The correlation between the charge generated by the MFCs with different catalysts for ORR and the COD of the AWW used to feed the devices was used to evaluate the biosensing capabilities of MFC‐based biosensors for the detection of total organic molecules in wastewater. Sensitivity, linear dynamic range, LOD, and response time of the MFC‐based biosensors were calculated as previously reported.

##### Statistical Analysis

Particle size and population were analyzed using SEM and TEM images with ImageJ software. The average particle size and population were determined by calculating the number of particles, and data were reported as mean ± standard error of the mean (SE). The SE was calculated using the formula $\frac{\text{SD}}{\sqrt{n}}$ where SD (standard deviation) was computed in Excel and *n* indicates the particle counted. All of the particles that were along the borders of the images which appeared as partial or incomplete structures were not counted within the analysis.

The current generated by each MFC biosensor was normalized by the projected anode area (12 cm^2^) to determine the current density. Statistical analysis was conducted in GraphPad Prism 10.4.1, with results presented as the mean of two MFC replicates for each catalyst ± SE.

The charge generated by each MFC biosensor was calculated as described in Equation (2), using a baseline of 10 μA. Statistical analysis of the calibration of the biosensor was performed using linear regression in GraphPad Prism 10.4.1. Data were presented as the mean of two MFC replicates for each tested catalyst ± SE.

## Conflict of Interest

The authors declare no conflict of interest.

## Author Contributions

**Alex Martinez Martin**: data curation (supporting); formal analysis (equal); investigation (equal); validation (lead); and writing—original draft (lead). **Shailza Saini**: data curation (lead); formal analysis (equal); investigation (equal); methodology (equal); and writing—review and editing (supporting). **Anna Salvian**: data curation (lead); formal analysis (equal); investigation (equal); methodology (lead); and writing—original draft (supporting). **Tarique Miah**: data curation (supporting) and formal analysis (supporting). **Cheuk Yiu Chan**: data curation (supporting) and formal analysis (supporting). **Claudio Avignone Rossa**: methodology (equal) and resources (equal). **Siddharth Gadkari**: conceptualization (lead); methodology (lead); resources (lead); supervision (lead); and writing—review and editing (lead). **Kalliopi Kousi**: conceptualization (lead); funding acquisition (lead); methodology (lead); project administration (lead); resources (lead); supervision (lead); and writing—review and editing (lead).

## Supporting information

Supplementary Material

## Data Availability

The data that support the findings of this study are openly available in [OPEN Research Surrey] at [https://doi.org/10.15126/surreydata.901566], reference number [901566].
