# Supplementary material for: Exsolution of Pt Nanoparticles from Mixed Zr/Gd‐CeO2 Oxides for Microbial Fuel Cell‐Based Biosensors
Source: Small Sci. 2025 Apr 1;5(6):2400619. doi: 10.1002/smsc.202400619 (PMC12168614; doi:10.1002/smsc.202400619)
Supplement: Supplementary file 1 — Supplementary Material [file SMSC-5-2400619-s001.pdf]

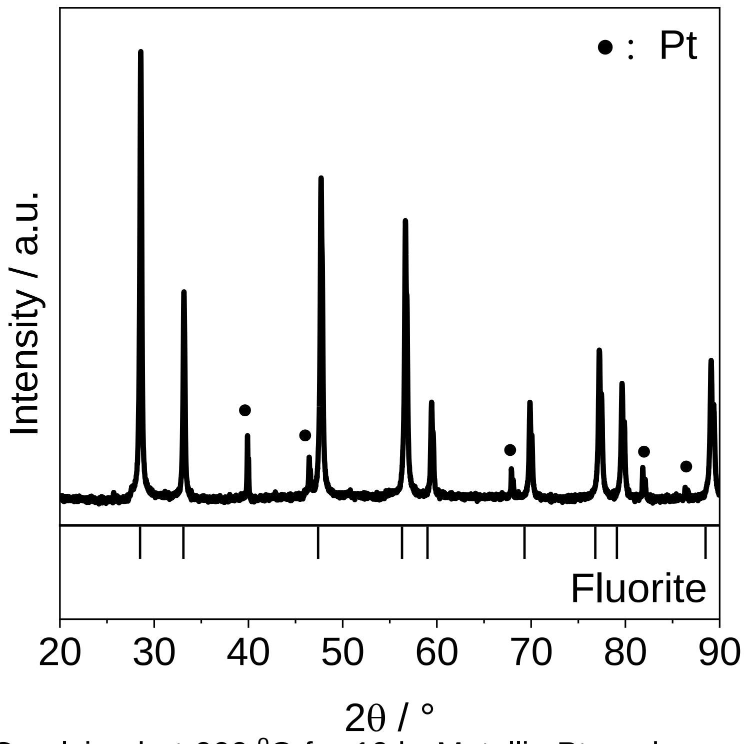

Figure S1. PCO calcined at 900 °C for 10 h. Metallic Pt peaks are observed and marked as ●.

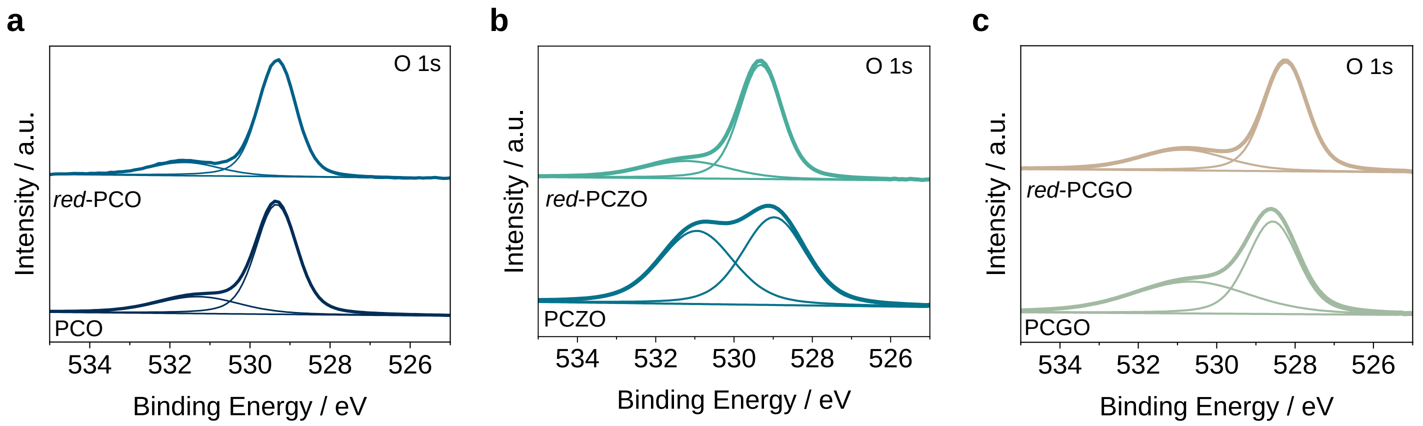

Figure S2. O 1s XPS spectra of **a**. PCO, **b**. PCZO, **c**. PCGO. Each image is depicting two different spectra. Bottom ones correspond to *as sintered* materials. Top ones correspond to the materials after undergoing reduction.

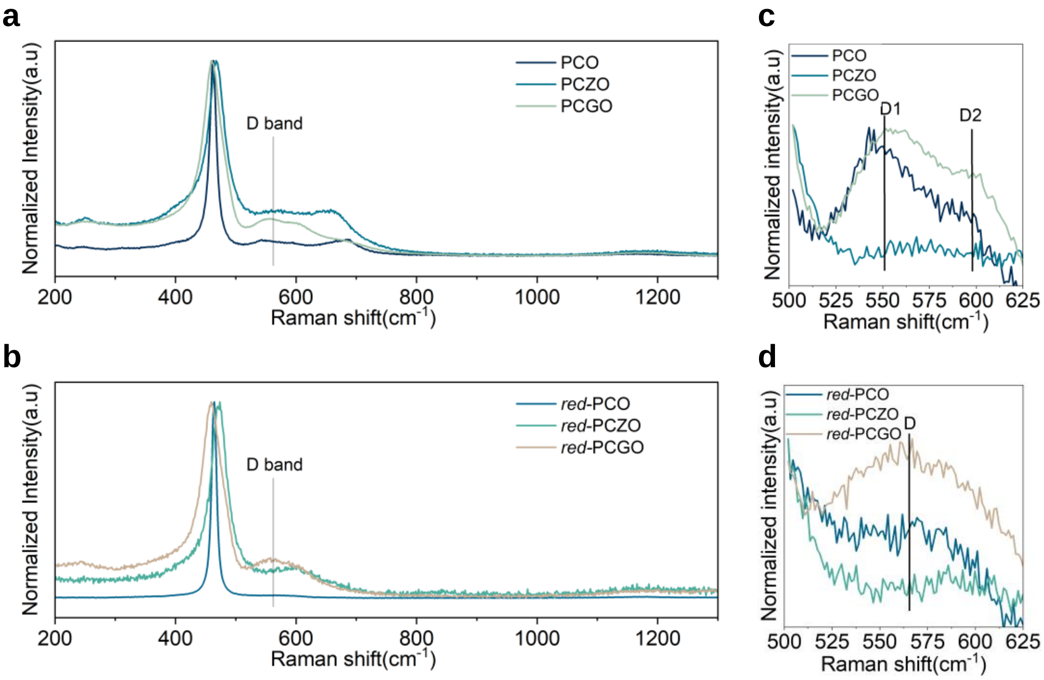

Figure S3. **a**,**b**. Raman spectrum of samples before and after reduction. **c**,**d**. Raman spectrum in the enrgy range 500-625 cm^{-1} linked to oxygen vacancies.

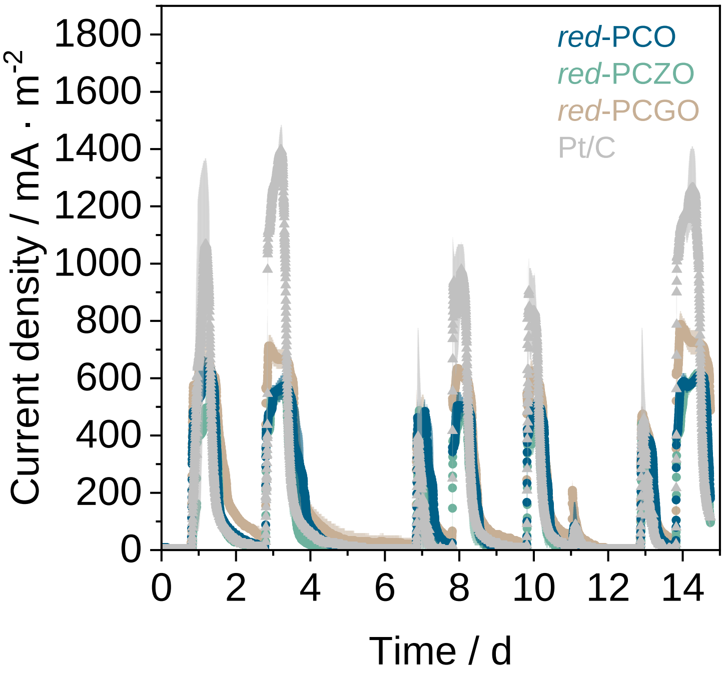

Figure S4. Calibration curve of MFC-based biosensors with the three different catalysts tested and the Pt/C electrode, as the one included in the original manuscript. Data are presented as the averages of two MFC replicates for each catalyst tested ± standard error of the mean (n=2). X-axis standard deviation bars are not visible as they are smaller than the symbols.

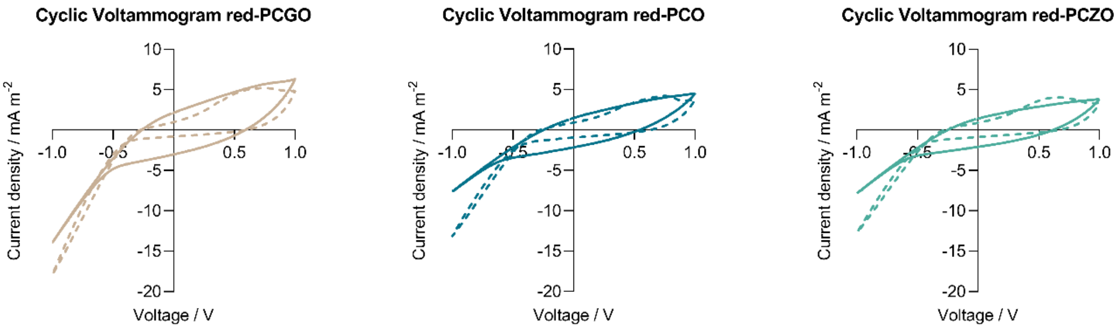

Figure S5. Chronoamperograms of the air-cathodes at the beginning of the experiment (t = 0 d, dotted line) and after 14 days of operation of the biosensor with AWW (t = 14 d, continuous line).

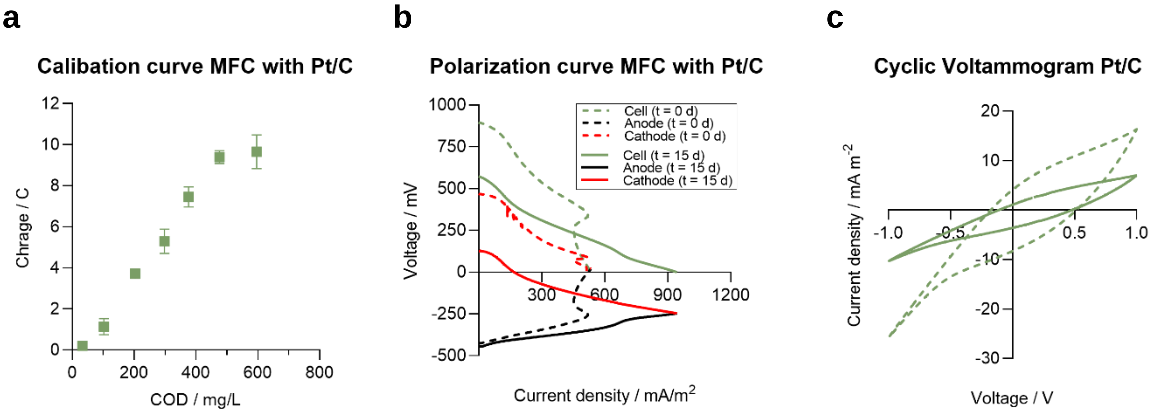

Figure S6. **a**. Calibration curve of MFC-based biosensor using standard Pt/C catalyst for ORR at the cathode. Data are represented as the average charge produced by two MFC replicates ± standard error of the mean (n=2). Error bars on the right axis are not visible as they are smaller than the symbols. **b**. Polarization curves of the cell, anode and cathode of MFC-based biosensor with standard Pt/C catalyst at the beginning of the experiment (t = 0 d) and after 14 days of operation with AWW (t = 14 d). **c**. Cyclic voltammogram of the cathode with standard Pt/C catalyst at t = 0 d (dotted line) and t = 14 d (continuous line).

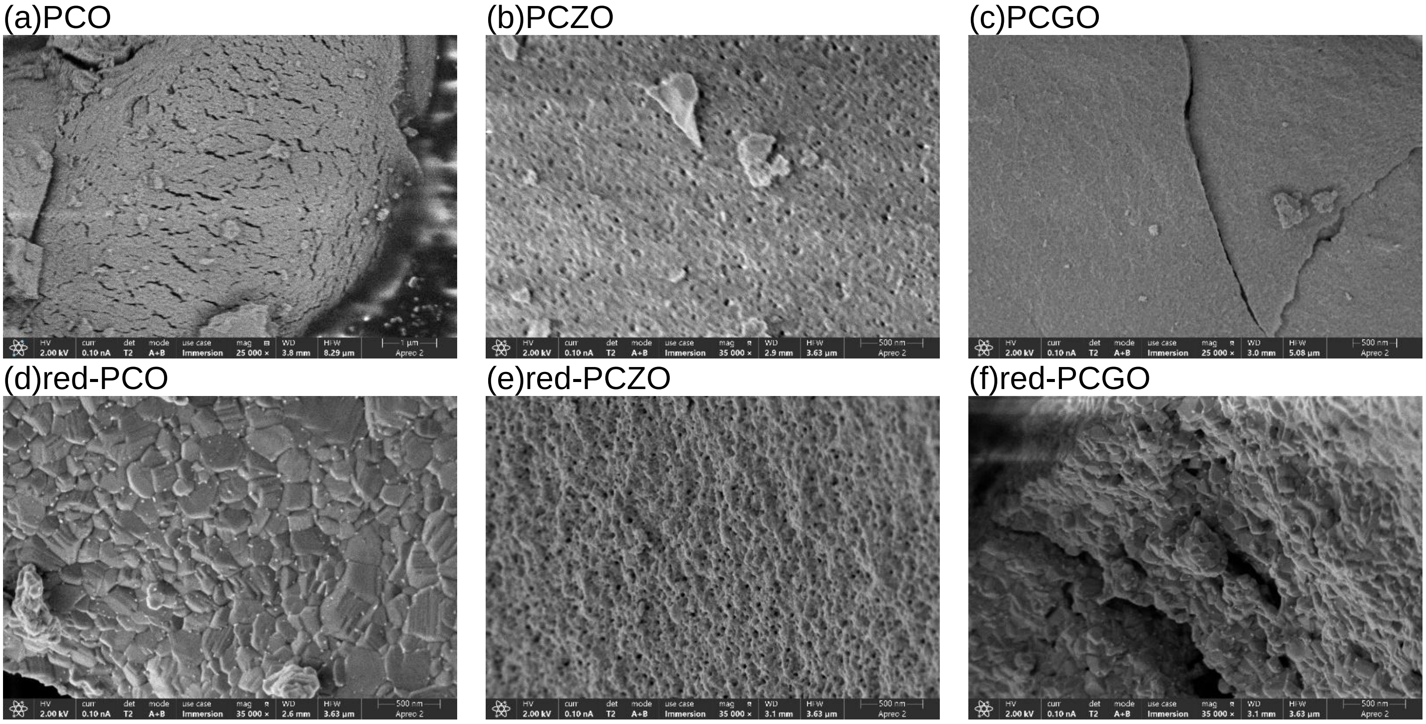

Figure S7. Additional SEM images of the fresh and reduced samples. Top ones correspond to the as sintered materials. (a) PCO, (b) PCZO (c) PCGO. Bottom ones correspond to the materials after reduction with Pt exsolution. (d) *red*-PCO, (e)*red*-PCZO, (f)*red*-PCGO

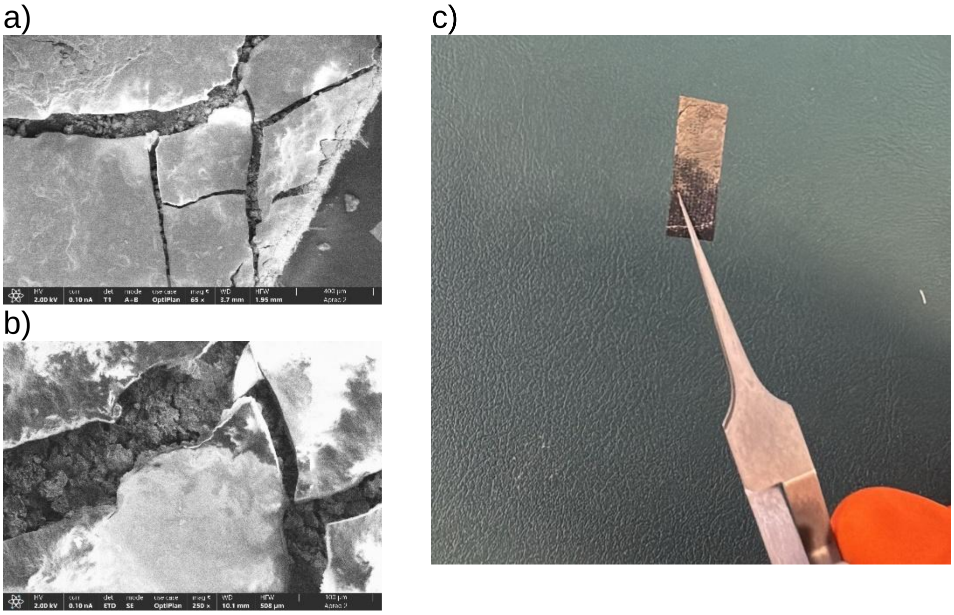

Figure S8. a,b. SEM images after testing for red-PCGO sample, c. Image of the sample covered with biological layer

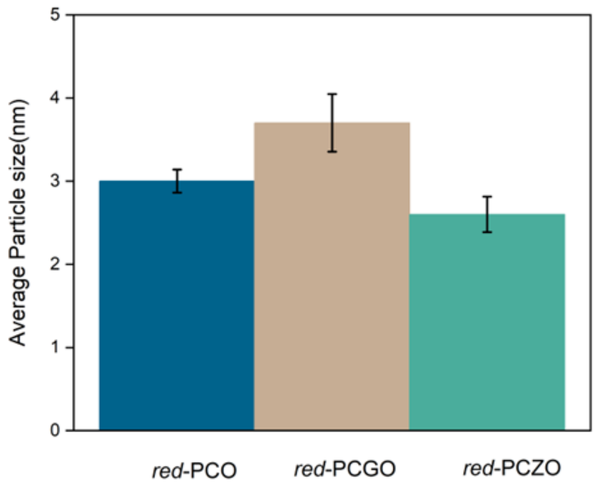

Figure S9. Average particle size of exsolved Pt after reduction. Data are represented as the average of Pt particle size from TEM images ± standard error of the mean (n=20). For *red*-PCO= 3.0 ± 0.1, *red*-PCGO= 3.7 ± 0.3, *red*-PCZO=2.6 ± 0.2

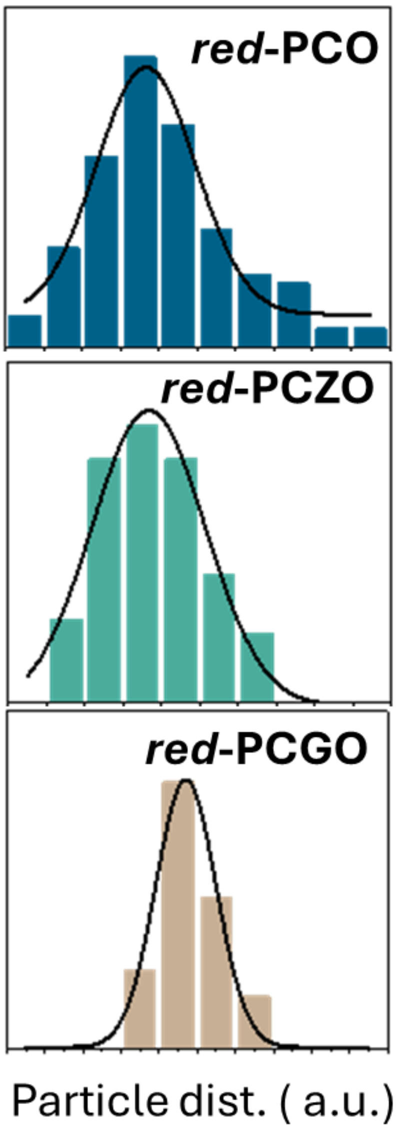

Figure S10. Particle size distribution of reduced samples. The particle count for the size distribution analysis is as follows: red-PCO (n = 250), red-PCZO (n = 220), and red-PCGO (n = 62). Note: This figure intends to illustrate the distribution and facilitate comparison between samples rather than provide absolute values, hence the absence of numerical values on the x- and y-axes.
